# Optimizing Biotinidase Activity Assays: Insights From Clinical and Laboratory Evaluation From a Low-Middle Income Country

**DOI:** 10.7759/cureus.78529

**Published:** 2025-02-04

**Authors:** Hafsa Majid, Muhammad Umer Naeem Effendi, Lena Jafri, Azeema Jamil, Bilal Hashmi, Halima Sarwar, Nasir A Khan, Sidra Ghori, Aysha Habib Khan

**Affiliations:** 1 Pathology and Laboratory Medicine, Aga Khan University, Karachi, PAK; 2 Clinical Laboratories, Aga Khan University, Karachi, PAK

**Keywords:** biotinidase, biotinidase deficiency, diagnosis, dried blood spot, newborn screening

## Abstract

Objective

This study aimed to verify the methods used for biotinidase deficiency (BTD) assays, including fluorometric and colorimetric techniques, measure biotinidase (BT) activity in dried blood spots (DBS) and serum samples, and explore the clinical spectrum of patients with BTD based on low serum BT activity.

Methods

A cross-sectional study was conducted at the Newborn Screening Lab, Aga Khan University, Karachi, following ethical approval from August 2021 to December 2024. The study was conducted in three phases. Phase 1 consisted of verification of performance characteristics (precision, accuracy, analytical measurement range, and linearity), according to Clinical Laboratory Improvement Amendments standards, of DBS-BT activity using a fluorometric enzyme immunoassay method. In phase 2, a colorimetric assay verified the performance characteristics of serum BT activity. Clinical evaluation of serum BT was assessed using data collected from 2021 to 2024 in phase 3.

Results

Phase 1: Precision of the DBS-BT assay, performed using level one and two controls, was acceptable, with a low coefficient of variation (CV) of 5.6%. Accuracy with proficiency specimens showed 100% agreement and an excellent correlation (r = 0.98). Phase 2: Precision of serum BT assay, performed using level one control, was acceptable, with a low CV of 4.86%. Accuracy with proficiency specimens showed 100% agreement and a correlation of r = 0.97. Phase 3: Clinical performance of DBS-BT samples (n = 438) and serum BT samples (n = 228) were conducted during the third phase. Clinical evaluation of serum BT samples revealed that 12.7% (n = 29) showed low BT activity and were diagnosed with BTD. These cases (82%, n = 23) were diagnosed after the neonatal/infantile period. In these patients, seizures were the most common clinical symptom (62%, n = 18), and high consanguinity was observed in 15 (51.7%) patient families.

Conclusion

These findings highlight the robustness of both DBS and serum BT tests in terms of precision, accuracy, and linearity. For diagnosing BTD, serum BT showed a good clinical detection rate. The delayed diagnosis and high consanguinity were noted, which emphasizes the need for enhanced screening programs and awareness, particularly in populations with high rates of consanguineous marriages.

## Introduction

Biotinidase deficiency (BTD) is a rare autosomal recessive metabolic disorder characterized by either a complete or partial deficiency of the enzyme biotinidase (BT), which is essential for recycling biotin [1]. Biotin serves as a cofactor for several carboxylase enzymes critical for gluconeogenesis, fatty acid synthesis, and amino acid catabolism [2]. A deficiency in BT disrupts the recycling of biotin, leading to impaired metabolic processes and the accumulation of toxic intermediates. While the global prevalence of BTD varies across populations, it is more common in regions with higher rates of consanguinity [3]. Newborn bloodspot screening programs have played a pivotal role in the early detection and management of this condition, significantly reducing associated morbidity and mortality [4].

BTD presents as either profound or partial deficiency, depending on the residual BT activity. Profound BTD, with less than 10% of normal enzymatic activity, typically manifests early infancy with severe neurological and dermatological symptoms such as seizures, hypotonia, developmental delay, hearing loss, optic atrophy, alopecia, and skin rashes [5]. Partial BTD, characterized by 10-30% of normal activity, is usually asymptomatic under normal conditions but may lead to symptoms under physiological stress, such as infection or prolonged fasting [6]. Timely diagnosis and supplementation with biotin are essential for the effective management of BTD [7].

Newborn screening (NBS) serves as the initial step in the diagnostic pathway for BTD, enabling the early detection of markedly reduced BT activity in dried blood spots (DBS). When NBS results indicate a potential deficiency, confirmatory testing is performed using a serum BT activity assay to classify the condition as either profound or partial deficiency. If biochemical testing confirms reduced enzyme activity, molecular genetic analysis of the BTD gene is undertaken to identify pathogenic variants and confirm the diagnosis [8].

Incorporating BT activity testing as a confirmatory step for positive NBS results in the Pakistani population is essential due to the high prevalence of consanguinity [9]. This approach enables accurate classification of profound and partial BTD, facilitating timely intervention and reducing the risk of severe, irreversible symptoms in affected infants. The goals of our study were to evaluate the method performance characteristics of DBS and serum BT and explore the clinical spectrum of patients with BTD based on low serum BT activity.

## Materials and methods

This cross-sectional study, conducted in three phases, was carried out in the Newborn Screening Lab, Section of Chemical Pathology, Department of Pathology and Laboratory Medicine, Aga Khan University, from August 2021 to December 2024. Optimization of DBS and serum BT were performed. Also, a clinical evaluation with serum BT was done. Approval from the institutional ethical review committee was taken before the commencement of the study (ERC ID: 2021-6008-17335).

Phase 1: optimization and performance characteristics verification of DBS-BT activity

Biochemical Analysis

It was assayed using the fluorometric enzyme immunoassay method using the commercial kit of LabSystem Diagnostic. It is based on monitoring fluorescent signals of the 6-aminoquinoline (6-AQ), a product formed during the reaction as a result of the BT activity. The BT enzyme in the blood sample utilizes biotin-6-AQ as a substrate. During the enzymatic reaction, this substrate is cleaved to a fluorescent product of 6-AQ, and biotin is produced. The reaction is terminated by adding ethanol to the wells, and the fluorescence in each well is measured with a microplate fluorometer on the Varioskan LUX Spectrofluorometer (Thermo Fisher Scientific, Waltham, MA) at 355 nm and 460 nm wavelengths. Two control levels are analyzed with every batch of samples. 

Performance Characteristics

For analytical validation of the DBS-BT assay, performance characteristics, including accuracy, precision, analytical measurement range (AMR), and linearity, were assessed according to Clinical Laboratory Improvement Amendments (CLIA) standards.

The precision of the assay was validated by evaluating both inter-day and intra-day variability by analyzing the manufacturer-provided controls in quadruples for five consecutive days to get 20 measurements or test results.

For accuracy (trueness) of DBS-BT, 15 proficiency testing specimens from the Centers for Disease Control and Prevention (CDC) were analyzed. Each year, 15 DBS samples are received from the CDC (three cycles and five specimens in each cycle) for proficiency testing of the DBS-BT assay.

For the linearity and AMR of DBS-BT, calibrators with concentrations of 13.2, 30.7, 50.7, 94.6, 181, and 381 U/L from six manufacturers were analyzed. All DBS-BT specimens were run in triplicates.

Phase 2: optimization and performance characteristics verification of serum BT activity

Biochemical Analysis

A method by Wolf et al. was followed [10]. Biotin-4-amidobenzoic acid was used as a substrate. All necessary reagents for this assay were commercially available but required individual preparation and validation before use. In this procedure, the serum was incubated with a buffered substrate (200 µmol of potassium phosphate) at 37°C for 30 minutes. The reaction was halted using 0.2 mL of 30% trichloroacetic acid. During incubation, BT in the patient's serum catalyzes the release of free p-aminobenzoic acid from the synthetic substrate. The liberated p-aminobenzoic acid undergoes diazotization with sodium nitrite, with excess nitrite subsequently removed using ammonium sulfamate. Finally, the diazotized p-aminobenzoic acid reacts with N-(1-naphthyl)ethylenediamine dihydrochloride to produce a mauve-colored product, which was measured spectrophotometrically at 546 nm. The measured absorbance directly correlates with the amount of p-aminobenzoic acid released, reflecting the BT activity present in the sample. Human BT enzyme activity in serum was determined using Colorimetry immunoassay on the Varioskan LUX Spectrofluorometer from Thermo Fisher Scientific.

Performance Characteristics

For analytical validation of the serum BT assay, performance characteristics, including accuracy, precision, AMR, linearity, and analytical sensitivity or limit of quantification (LOQ), were assessed.

The precision of the assay was determined using normal and abnormal levels of control materials. Serum samples of a healthy volunteer were collected in a gel top container, centrifuged at 3,000 rpm for 10 minutes, and saved at -80°C until analysis. Abnormal or positive serum control, representing decreased BT activity, was prepared by heat inactivating a large pool of normal serum at 60°C for one hour as recommended by the American College of Medical Genetics and Genomics [8].

For accuracy (trueness), 16 proficiency testing specimens by the European Research Network for Diagnosis of Inherited Metabolic Disorders (ERNDIM) received in 2022 and 2023 were analyzed in duplicates. Eight ERNDIM specimens of serum BT are received each year for proficiency testing.

For the linearity and AMR, a stock standard of 33.3 U/L was made, and 1:1 and 1:10 dilutions of this stock solution were made to achieve concentrations of 16.67 U/L and 3.33U/L. A blank specimen was also analyzed along with the three mentioned concentration serum specimens.

For serum BT, LOQ was also analyzed with two samples, a blank and a calibrator of 3.33 U/L, which were analyzed 10 times each to determine the LOQ.

Phase 3: clinical performance evaluation of DBS-BT and serum BT assays

Clinical performance was assessed using the data of the patients analyzed for DBS and serum BT assays from January 2021 to December 2024 and January to December 2024, respectively. Normal DBS and serum BT activity for a healthy newborn is >60 nmol/minute/dL and >3.5U/L, respectively. Data on the DBS and serum BT performed was extracted from the Laboratory Information System, while the clinical history of positive cases was collected from their parents using a structured questionnaire (Appendices) after obtaining verbal informed consent via telephone.

Statistical analysis

Microsoft Excel (Microsoft® Corp., Redmond, WA) and EP Evaluator software (Data Innovations, São Paulo, Brazil) were used for data analysis. For categorical data, frequency and percentages were used, while for quantitative data, median and quartiles were reported. For precision assessment, means, standard deviation (SD), and coefficient of variation (CV) were calculated using the formula (SD/mean × 100). The accuracy of DBS and serum BT was assessed using Deming regression and slope; the intercept and correlation coefficient (r) were measured. Recovery was calculated for AMR and linearity, while mean and SD were compared against manufacturer claims to assess LOQ.

## Results

Phase 1: performance characteristic verification of DBS-BT

**Figure 1:**
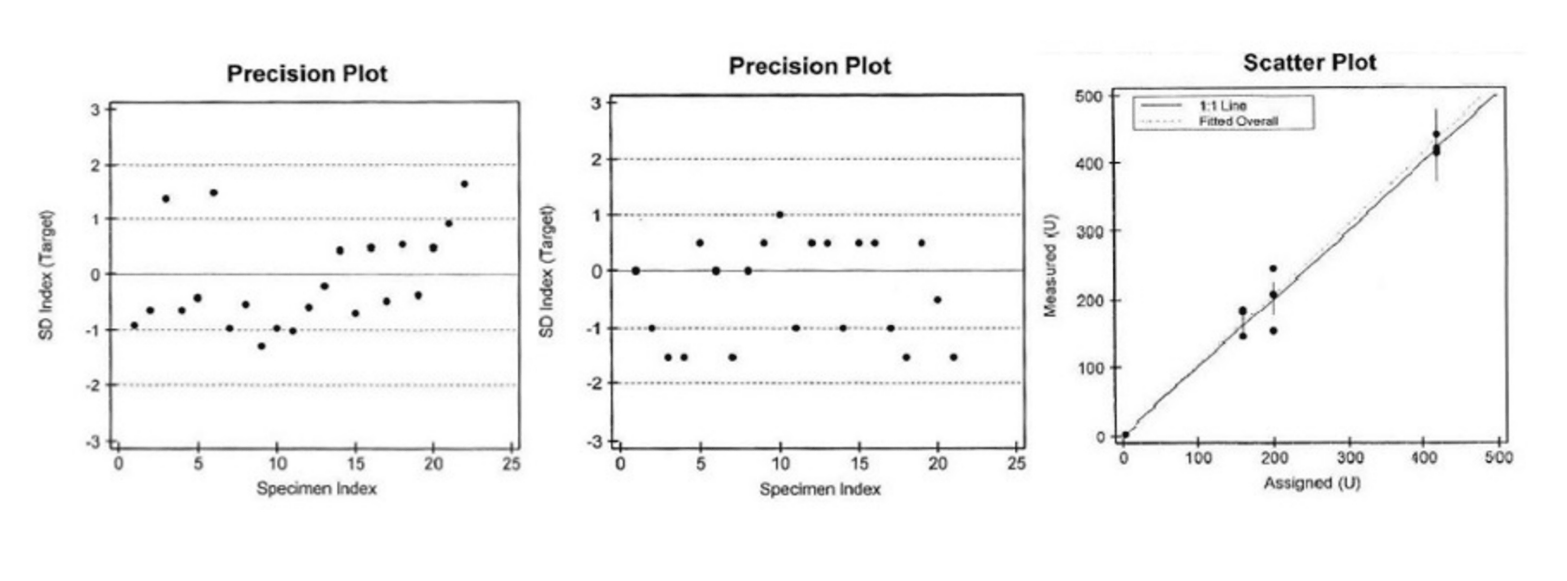
Precision plot of level 1 (a), level 2 (b) control, and scatter plot (c) showing accuracy, linearity/AMR verification of DBS-BT assay. AMR, analytical measurement range; DBS-BT, dried blood spots-biotinidase

Precision of two-level controls, L1 and L2, was done. The observed mean, SD, and CV for both L1 and L2 were less than the manufacturer claimed to mean, SD, and CV; hence, they were acceptable, as shown in Table 1. The precision plots of L1 and L2 are shown in Figures 1a-1b, respectively.

**Table 1:** Experimental results of precision study using levels 1 and 2 controls for DBS-BT assay. CV, coefficient of variation; DBS-BT, dried blood spots-biotinidase; SD, standard deviation

Statistics		Observed	Manufacturer-claimed
Level 1 control	Mean (U/L)	31.3	32
	SD	1.8	2.0
	CV (%)	5.6	6.3
Level 2 control	Mean (U/L)	299	301
	SD	16.5	18.6
	CV (%)	5.5	6.2

For accuracy, out of the 15 specimens analyzed (six with low BT activity), they showed 100% agreement, and on linear regression analysis, correlation, slope, and intercept of 0.98, 1.03, and 0.01, respectively. The experimental results of AMR/linearity are shown in Table 2 and were considered acceptable. A scatter plot showing accuracy and linearity/AMR verification of the DBS-BT assay is shown in Figure 1c.

**Table 2:** Experimental results of linearity study using four standards for DBS-BT assay. DBS-BT, dried blood spots-biotinidase

Standards (U/L)		Measured concentrations (U/L)			Mean (U/L)	Recovery (%)	Linearity
S1	3	3	3.1	3	3.03	101	Pass
S2	160	144	184	181	169.7	106	Pass
S3	200	245	208	152	201.7	100.8	Pass
S4	420	414	440	419	424.3	101	Pass

Phase 2: performance characteristic verification of serum BT

**Figure 2:**
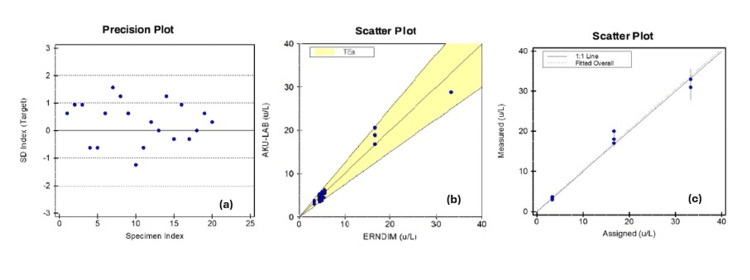
Precision plot (a) of normal control, scatter plots showing accuracy (b), and linearity/AMR (c) verification of serum BT assay. AMR, analytical measurement range; BT, biotinidase

Precision of normal control was done. The observed and manufacturer-claimed mean, SD, and CV were 5.10 U/L, 0.24 U/L, 4.86%, 5.0 U/L, 0.32 U/L, and 6.3%, respectively. The observed mean, SD, and CV were 5.1 U/L, 0.24, and 4.86%, while the manufacturer's claimed mean, SD, and CV were 5.0 U/L, 0.32, and 6.3%, respectively. The observed values were lower than manufacturer-claimed values; hence, the precision study was acceptable, as shown in Figure 2a.

For accuracy, 11 ERNDIM proficiency testing specimens were analyzed, and on analysis, 100% agreement was noted with a Cohen's kappa value of 1, whereas linear regression analysis revealed a slope of 0.976 (confidence interval (CI) 0.905-1.047), a correlation coefficient of 0.97, and an intercept of 0.12 (range -0.49 to 0.74), as shown in Figure 2b.

The experimental results of AMR/linearity are shown in Table 3 and were considered acceptable (Figure 2c). The analytical sensitivity, measured using a blank and low concentration standard, was deemed acceptable. 

**Table 3:** Experimental results of linearity study using three standards for serum biotinidase assay.

Standards (U/L)		Measured concentrations (U/L)			Mean (U/L)	Recovery (%)	Linearity
S1	3.33	3.3	3.0	3.7	3.33	100.1	Pass
S2	16.67	17	18	20	18.33	110	Pass
S3	33.33	31	31	33	31.67	95.1	Pass

Phase 3: clinical performance of DBS-BT and serum BT

A total of 438 subjects were tested for DBS-BT over the study period. Of these, low DBS-BT activity was noted in 13 (2.96%). The demographic characteristics of the subjects tested for DBS-BT are shown in Table 4. Six (out of the 13) patients with low DBS-BT activity were further tested for serum-BT activity, and all had normal activity.

**Table 4:** Demographic characteristics of patients tested for DBS and serum BT activity.

Characteristics		Patients tested for serum BT activity (U/L)		Patients tested for DBS-BT activity (nmol/minute/dL)	
		All patients (n = 228)	Patients with low activity (n = 29)	All patients (n = 438)	Patients with low activity (n = 13)
Gender	Male	135 (59.2%)	18 (62%)	268 (61.2%)	7 (53.8%)
	Female	93 (40.8%)	11 (38%)	170 (38.8%)	6 (46.2 %)
Median (Q3-Q1) age in years		0.92 (2.5-0.5)	1.43 (3-0.44)	1.04 (2.89-0.44)	0.16 (0.45-0.01)
Median (Q3-Q1) BT levels in U/L		11.5 (15.7-7.47)	<3.5	191 (254.6-125.2)	44.9 (55.2-22.2)

**Figure 3:**
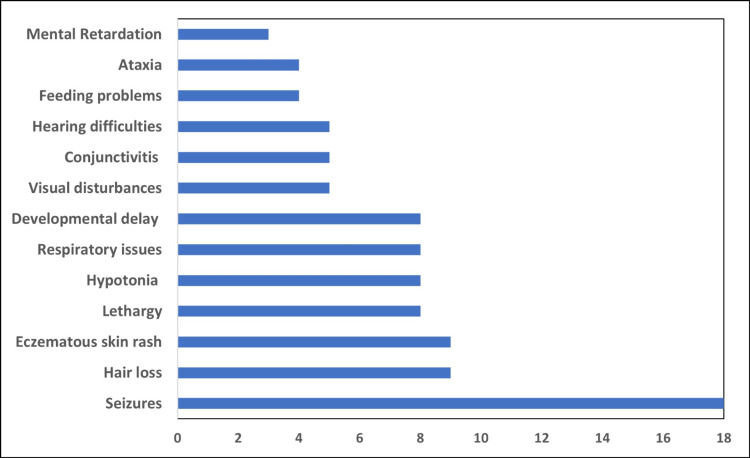
Clinical spectrum in patients with biotinidase (BT) deficiency based on low serum BT activity (U/L).

A total of 228 subjects were tested for serum BT over the study period. Of these, 29 (12.7%) exhibited low serum BT activity (<3.5 U/L) and were diagnosed with BTD (Table 4). In these patients, seizure was the most common clinical finding, noted in 18 (62%) of the patients, followed by eczematous skin rash and hair loss or alopecia, noted in nine (31%) patients each (Figure 3). Of the 29 patients, consanguineous marriage was noted in 15 (51.7%) patient families; siblings of two (7%) of the patients were also diagnosed with BTD, while siblings of another five (17%) patients had symptoms similar to BTD. Among the 29 patients with BTD, only five and one presented during infantile and neonatal periods, respectively. At the same time, 23 (82%) were diagnosed after one year of age.

## Discussion

DBS and serum BT activity assays are used for screening and diagnosing BTD. This is an autosomal recessive disease, which is particularly prevalent in populations with high consanguinity, such as Pakistan. However, optimization of these assays is important prior to BTD’s inclusion in NBS programs to ensure accurate diagnosis. In this study, performance characteristics of DBS and serum BT activity assays were rigorously verified. Both assays demonstrated robust precision, accuracy, and linearity, confirming their reliability for clinical use.

During the study, 438 newborns were tested for DBS-BT, and 13 (2.96%) were screened positive for BTD. The affected individuals had a median age of 0.16 years (0.45-0.01) and a median DBS-BT activity of 44.9 nmol/minute/dL (55.2-22.2). In comparison, Gopalakrishnan et al. identified 22 newborns with low DBS-BT activity among 1,680 screened in Uttar Pradesh, India [11]. Similarly, Semeraro et al. analyzed data from the first three years of NBS for BTD in Abruzzo, Italy, and identified 16 cases out of 26,393 screened newborns [12]. In another study, Neto et al. reported absent or low BT activity in 272 newborns among 225,136 screened in Brazil [13]. This difference could be explained by the higher prevalence of consanguinity in Pakistan, which increases the likelihood of autosomal recessive disorders such as BTD. Other contributing factors may include cutoff thresholds for defining low activity and variations in population characteristics, such as genetic diversity.

During the study period, 228 individuals were tested for serum BT, among whom 29 (12.7%) were found to have low activity. The majority of these cases were male, accounting for 18 (62%) patients. The median age of subjects with low serum BT activity was 1.43 years (3-0.44). In comparison to our findings, a study by Lara et al. confirmed BTD in 10 out of 129 suspected newborns using quantitative serum testing [14]. While both studies underscore the importance of confirmatory serum BT testing, our study reports a higher detection rate, potentially reflecting differences in population characteristics or genetic factors such as higher consanguinity rates in our cohort. Seizures were the most frequently observed clinical feature, occurring in 18 (62%) patients. Similar findings have been reported in other studies. For instance, a case series from Malaysia screened clinically suspected patients for BTD using fluorometric methods and observed seizures in more than 70% of affected children [15]. This pattern is further supported by several original studies and review articles, underscoring the prevalence of seizures as a prominent feature in this condition [16-18].

In the context of Pakistan and other low-middle-income countries, these findings highlight a concerning delay in the diagnosis of BTD, with most cases being identified well beyond the critical neonatal and infantile periods when irreversible neurological damage has already occurred. This delay can likely be attributed to the absence of robust NBS programs in the country and also limited awareness about the condition among healthcare providers. The high rate of consanguineous marriages further exacerbates the situation, increasing the likelihood of inherited metabolic disorders, including BTD. Moreover, the prominence of seizures as a presenting symptom underscores the importance of early recognition and testing in children with neurological symptoms to prevent severe complications. These observations emphasize an urgent need to prioritize awareness campaigns, genetic counseling, and the establishment of accessible diagnostic and screening facilities tailored to the regional healthcare landscape.

The study has some limitations that need to be acknowledged. All subjects who tested positive for DBS-BT were not confirmed by testing for serum BT activity, limiting the identification of true BTD cases. Also, the positive and negative predictive value of DBS-BT could not be evaluated due to the cross-sectional study design. This warrants further investigation in larger and longitudinal studies.

## Conclusions

The study validated the DBS and serum BT assays in a population of a low-middle country with high consanguinity. The primary results reveal that the use of DBS assay provides a reliable screening tool, hence ensuring the accuracy of DBS and serum assay for the screening and diagnosis of BTD in contexts limited by resources, which is crucial for preventing serious neurological results. The age of presentation was late, and neurological symptoms were more prevalent. These findings necessitate that BTD should be included in the national NBS program, especially in low-middle countries with high consanguinity. This will help in early detection and treatment initiation and alleviate the burden on the health systems of low-middle countries.

## Appendices

**Table 5:** Questionnaire for biotinidase deficiency patients. Credits: Hafsa Majid, Muhammad Umer Naeem Effendi, Lena Jafri, Azeema Jamil, Syed Bilal Hashmi, Halima Sarwar, Nasir Ali, Sidra Ghori, Aysha Habib Khan

Demographics	
Study ID:	
Age (years):	
Date of sampling:	
MR#/L#:	
Gender □ Male □ Female	
Referred by:	
Clinical history	
Sign and symptoms	(√ if present)
Lethargy/floppiness	
Seizures	
Hypotonia	
Eye changes	
Hair loss	
Conjunctivitis	
Respiratory issues	
Developmental delay	
Mental retardation	
Feeding problems	
Hearing difficulties	
Eczematous skin rash	
Ataxia	
Other	
Family history	
Question	(√ if present)
Does parents have cousin marriage?	
No. of siblings	
Any other sibling had similar symptoms. If yes, which siblings were they tested? What were the results?	
Were there any unexplained deaths of siblings? If yes, what was their age? ___. Cause: __________	
Any maternal/paternal relative who has similar symptoms	
Do the parents have similar symptoms?	
Are siblings normal for their age?	
If not, do they have any neurological disability? Seizures/developmental delay/mental retardation	
Other diseases identified in siblings. If yes, what? _____________	
